# The Challenges of Recruiting and Retaining Dyads of Hospitalized Persons With Dementia and Their Family Carers

**DOI:** 10.1093/geroni/igaa057.2733

**Published:** 2020-12-16

**Authors:** Marie Boltz, Ashley Kuzmik

**Affiliations:** 1 Penn State University, University Park, Pennsylvania, United States; 2 Pennsylvania State University, University Park, Pennsylvania, United States

## Abstract

Persons with dementia (PWD) have high rates of hospitalization, and along with their family caregivers (FCGs), commonly experience negative hospital experiences and outcomes. The recruitment and retention challenges encountered in an ongoing cluster randomized clinical trial in PWDs and FCGs are described. The trial tests the efficacy of a nurse-FCG partnership model that aims to improve: 1) the physical and cognitive recovery in hospitalized PWD, and 2) FCG preparedness and anxiety. Recruitment and retention challenges, identified in team meetings and extracted from team documentation,.include factors in the hospital environment, the PWD, and FCGs. Strategies that address these challenges include careful pre-planning and preparation with the site, strong communication with dyads, and honoring preferences for communication. The recruitment and retention of acutely ill older adults with dementia and FCGs can pose a challenge to investigators and threaten the validity of findings. Recruitment and retention strategies that help improve validity are described

